# Sexual Health Encounters: Competence and Barriers Among Family Medicine Residents and Primary Care Physicians of the National Guard in the Western Region of Saudi Arabia

**DOI:** 10.7759/cureus.76777

**Published:** 2025-01-02

**Authors:** Sameer Rizg, Sultan A Almuntashiri, Saleh Alamri, Sultan Alsehli, Hamzah Alharbi, Abdulelah Gadah

**Affiliations:** 1 Family Medicine, King Abdulaziz Medical City, Jeddah, SAU

**Keywords:** physician competence level, primary health care, sexual health, sexual health barriers, sexual health issues

## Abstract

Aim: The study aimed to survey the self-reported competence of primary care physicians in the National Guard in addressing and treating sexual health issues at King Abdulaziz Medical City Primary Health Care (PHC) centers in the Western Region of Saudi Arabia.

Method: In this cross-sectional study, the region was represented by three cities as follows: three PHC centers from Jeddah City (Iskan PHC Center (King Faisal Residential City), Specialized Poly Clinic PHC Center, and Bahra PHC Center at National Guard Hospital), one PHC center from Taif City (Iskan PHC Center (King Khalid Residential City)), and one PHC center from Makkah City (Al-Sharaie PHC Center). A total of 93 participants were included in this study.

Results: This study revealed that nearly half of the participants (40, 43%) reported having moderate levels of perceived competence, while nearly half (39, 41.9%) noted moderate barriers to addressing sexual health issues.

Conclusion: Evidence of moderate levels of perceived competence and barriers suggests the need to address sexual health education.

## Introduction

Sexual health is essential in life; sexual and reproductive health is defined as "a state of physical, emotional, mental, and social well-being and not simply the absence of disease or infirmity" [1].

Sexual healthcare assessment is conducted in different healthcare settings, including primary health settings, in which it comes in different presentations, including sexually transmitted diseases (STDs), pregnancy assessment with antenatal care, fertility issues, and sexual dysfunction [2].

Sexual health assessment, evaluation, and management are important as they are related to improving quality of life. A study found that 50% of male participants and 40% of female participants stated that there is a relationship between good sexual health and an increase in quality of life [3]. However, due to the specific nature of sexual issues, patients may have difficulties addressing their sexual problems with their doctors [4]; hence, the importance of physicians taking the lead in addressing such sexual health problems with their patients. Moreover, general practitioners (GPs) and family medicine physicians are considered the front line in the healthcare system. For some patients, the primary healthcare provider is the only accessible doctor from whom they can seek help regarding all health issues [5].

STDs are increasing worldwide, with more than one million patients becoming infected daily, most of whom are asymptomatic [6]. In Riyadh, Saudi Arabia, a study conducted on 225 first-year college students found that 31% of them engaged in sexual activity at least once before being married, and more than 50% of them were not knowledgeable about condom use as a way to prevent STDs. Further, 20% of the students were not aware that human immunodeficiency virus (HIV) can be transmitted through both homosexual and heterosexual contact [7].

A large number of sexual issues are undertreated in primary health care (PHC), and managing STDs appears to be easier than dealing with and addressing issues related to sexual functionality [8]. Moreover, sexual health issues seem to be complex and have multiple barriers that can be assessed and prevented, thus improving health care assessment, management, and prevention. The following sexual assessment barriers include lack of time during the visit, lack of training and knowledge regarding sexual health, dealing with the opposite gender, discussing sexual health with a patient from a different culture or religion, and discussing sexual history with teenagers or unmarried couples [2,9].

With this knowledge, physicians must be able to provide a proper approach to sexual health issues and screenings, educate the population regarding sexual health matters, and give them adequate counseling [10]. STDs remain a challenging issue, regardless of age, and addressing STDs in Saudi Arabia is considered taboo, where social norms and ethics pose many obstacles, such as social desirability bias and fear of judgment. People living in conservative societies are more likely to be denied STD information. As a result, there is little information about STD knowledge and views among these communities. Therefore, this study aimed to survey the self-reported competence of primary care physicians in the National Guard in addressing and treating sexual health issues at King Abdulaziz Medical City (KAMC) PHC centers in the Western Region of Saudi Arabia.

A cross-sectional study in Finland in 2020 assessed GPs’ self-competence, barriers in addressing sexual health, and educational needs in sexual medicine. It was found that 96% of GPs felt competent in discussing and treating sexual health issues. The main barriers were short appointment times and lack of knowledge and experience in sexual medicine. Gender differences emerged, with male GPs facing more barriers when dealing with opposite-gender patients, while female GPs reported the lack of effective treatment and fear of failing to respond to patients’ sexual health issues to hinder bringing up the subject. Medical journals and medical school education were key sources of knowledge, but 82.6% of participants found their education insufficient, and 87.8% expressed the need for more training in sexual medicine [9].

Another cross-sectional study in Sheffield, UK, involving 22 GPs and 35 practice nurses, identified key barriers to discussing sexual health in primary care, including time constraints, competing priorities, and gender preferences. Participants reported greater comfort with same-gender patients and noted that certain ethnic groups (e.g., Black, Asian, African-Caribbean, and Muslim) were less open to sexual health discussions. Age was also a factor, with individuals aged 40 and above perceiving sexual health as more personal and sensitive [11].

In Portugal, a cross-sectional study evaluated GPs’ knowledge, attitudes, and beliefs concerning sexual dysfunction (SD) practices, SD management, self-perceived competence, and training needs. It was found that more than 50% of the GPs reported that their medical degree was not an adequate source of training, and more than 90% reported the need for continued training. Major barriers included personal attitudes and beliefs, time constraints, lack of knowledge, experience, and training. A preference for the same gender was also identified, with GPs reporting low confidence in treating SD in opposite-gender patients [12].

In the USA, a cross-sectional study assessed the impact of physician gender on sexual history taking among obstetricians/gynecologists (OB/GYNs), family practitioners, internists, pediatricians, and surgeons. It was found that patients younger than 18 or older than 60, those with academic achievement below the college level, and those who were divorced or single reported discomfort. Physicians also reported discomfort when dealing with opposite-gender patients [13].

Another cross-sectional study in Staffordshire, UK, assessed GPs’ training needs in sexual health. It was found that 75% of GPs received less than five days of sexual health education in medical school and mainly relied on experience and self-teaching afterward. Most GPs believed their attitudes influenced their advice, with moral advice not being given or only sometimes given. All participants reported minimal education in sexual health and expressed a need for further training [14].

Another cross-sectional study in Malaysia assessed senior-year medical students' attitudes and training regarding sexual history taking. Most students acknowledged its importance, but half felt uncomfortable discussing sexual issues. Barriers included age, marital status, sexual orientation, and cultural or religious differences. Half of the students felt inadequately trained in sexual history taking during medical school [2].

## Materials and methods

This cross-sectional study was conducted at KAMC PHC centers, Western Region, Saudi Arabia. The region was represented by three cities as follows: three PHC centers from Jeddah City (Iskan PHC Center (King Faisal Residential City), Specialized Poly Clinic PHC Center, and Bahra PHC Center at National Guard Hospital), one PHC center from Taif City (Iskan PHC Center (King Khalid Residential City)), and one PHC center from Makkah city (Al-Sharaie PHC Center). Ethical approval for the study was obtained from the King Abdullah International Medical Research Center.

The sample size of each center was computed with a margin of error of 5% and a confidence level of 95%. Out of the initial recruitment of 143 participants, a minimum of 105 participants was required; 93 were ultimately included in the study. An inclusive consecutive sampling approach was employed, targeting all physicians on duty at the selected PHC centers during data collection. The inclusion criteria were family medicine physicians and residents from the Ministry of National Guard, Western Region, Saudi Arabia. Participants were excluded if they had incomplete data or refused to participate in the study.

The questionnaires were administered directly by the co-authors, who personally visited the PHC centers to ensure accurate data collection.

The study included a questionnaire with 23 questions adopted from a Finnish study by Manninen et al. [9], with permission granted by the main author. Informed consent forms were provided before the commencement of the research. The questionnaires were distributed to participants in printed form, with participants filling them out independently as self-administered questionnaires. To ensure accurate data collection, the questionnaires were administered directly by the co-authors, who personally visited the PHC centers. In cases of troubleshooting, one of the co-authors was available to assist with any difficulties in understanding. Participants’ information obtained by the researchers was protected from unauthorized personnel. The questionnaires were coded by number to keep the identities of participants anonymous. All of the gathered information was stored in a password-protected computer owned by the researchers.

The research study underwent three phases: (1) A preparatory period (four weeks), including title selection, literature review, research proposal, and securing ethical approval; (2) fieldwork (four weeks), including data collection through a self-administered questionnaire and data analysis with interpretation; and (3) finalizing the research (two to four weeks).

Data entry and analysis

Data were entered and analyzed using IBM SPSS Statistics for Windows, Version 23.0 (Released 2015; IBM Corp., Armonk, New York, USA). Continuous variables were presented as means and standard deviations (SD), and categorical variables were presented as frequencies and percentages. The chi-square test was used to compare two or more qualitative variables, Student's t-test was used to compare two independent quantitative variables, and the ANOVA test was used to compare more than two independent quantitative variables. Significance was determined at a p-value <0.05 and a 95% confidence interval (CI). Other appropriate statistical tests were used as needed.

## Results

Of 93 participants, 58 (62.4%) were female and 35 (37.6%) were male. Of these, 39 (41.9%) were residents, 22 (23.7%) were GPs, and both board-certified/associate consultants and consultants comprised 16 (17.2%) of the study population. The socio-demographics of the included participants are shown in Table 1.

**Table 1:** Demographics/characteristics of family medicine physicians and residents at KAMC PHC centers. GP: general practitioner; KAMC: King Abdulaziz Medical City; PHC: Primary Health Care.

Demographics		Count	%
Total		93	100.0
Gender	Male	35	37.6
	Female	58	62.4
Position	Consultant	16	17.2
	Board-certified/associate consultant	16	17.2
	GP	22	23.7
	Resident	39	41.9

Table 2 shows the percentage of perceived level scores among the participants regarding how they address the sexual health issues of patients. Each item had a four-point Likert scale with scoring of 0-3 depending on the answers: Not a problem = 3, A minor problem = 2, A moderate problem = 1, A major problem = 0; Good = 3, Quite good = 2, Quite poor = 1, Poor = 0; Always = 3, Usually = 2, Seldom = 1, and Never = 0. Most participants revealed high levels of perceived competence; however, nearly a third of them (31, 33.3%) showed “quite poor” levels when treating male patients’ sexual problems compared to females, with only 12 (12.9%). Moreover, 46 (49.5%) answered "seldom" to "never" when exploring patient satisfaction with their sexual health.

**Table 2:** Percentage of the perceived competence level of family physicians towards addressing sexual health issues (n = 93).

Perceived competence		Count	%
Total		93	100.0
How easy is it for you to discuss sexual health issues if your patient addresses the subject?	Not a problem	40	43.0
	A minor problem	32	34.4
	A moderate problem	17	18.3
	A major problem	4	4.3
How do you classify your competence in discussing sexual problems with male patients?	Good	28	30.1
	Quite good	32	34.4
	Quite poor	21	22.6
	Poor	12	12.9
How do you classify your competence in discussing sexual problems with female patients?	Good	43	46.2
	Quite good	31	33.3
	Quite poor	13	14.0
	Poor	6	6.5
How do you classify your competence in treating male patients’ sexual problems?	Good	28	30.1
	Quite good	23	24.7
	Quite poor	31	33.3
	Poor	11	11.8
How do you classify your competence in treating female patients’ sexual problems?	Good	45	48.4
	Quite good	31	33.3
	Quite poor	12	12.9
	Poor	5	5.4
When you take a sexual history, do you explore how satisfied the patient is with his/her sexual life?	Always	17	18.3
	Usually	30	32.3
	Seldom	30	32.3
	Never	16	17.2

Table 3 shows the percentage of perceived sexual health barriers among the participants regarding how they address the sexual health issues of patients. Each item had a 4-point Likert scale with scoring of 0-2 depending on the answers: Not at all = 0, Some = 1, Cannot say = 1, Much = 2, and Very much = 2. Among the listed perceived sexual health barriers, gender differences were considered the top barrier in addressing sexual health issues, with 44 (47.3%) reporting this. Personal discomfort was noted by 32 (34.4%), personal attitudes and beliefs by 31 (33.3%), and sexual health issues not being a priority in the appointment by 27 (29.0%).

**Table 3:** Percentage of perceived sexual health barriers of family physicians towards addressing sexual health issues (n = 93).

Sexual health issues barriers (n = 93)	Not at all	Some	Cannot say	Much	Very much
Shortness of the appointment time	16 (17.2%)	20 (21.5%)	8 (8.6%)	27 (29.0%)	22 (23.7%)
Sexual health issues not being a priority in the appointment	17 (18.3%)	21 (22.6%)	7 (7.5%)	27 (29.0%)	21 (22.6%)
Personal attitudes and beliefs	12 (12.9%)	18 (19.4%)	7 (7.5%)	31 (33.3%)	25 (26.9%)
Personal discomfort when addressing sexual health issues	10 (10.8%)	20 (21.5%)	1 (1.1%)	30 (32.3%)	32 (34.4%)
Lack of knowledge about sexual medicine	18 (19.4%)	25 (26.9%)	9 (9.7%)	21 (22.6%)	20 (21.5%)
Lack of experience with sexual medicine	10 (10.8%)	32 (34.4%)	7 (7.5%)	25 (26.9%)	19 (20.4%)
Lack of effective treatment	24 (25.8%)	28 (30.1%)	10 (10.8%)	25 (26.9%)	6 (6.5%)
Fear of failing to respond to patients’ sexual health issues	22 (23.7%)	27 (29.0%)	6 (6.5%)	28 (30.1%)	10 (10.8%)
Gender differences (where the patient represents the opposite gender)	5 (5.4%)	15 (16.1%)	5 (5.4%)	24 (25.8%)	44 (47.3%)
Disability of the patient	22 (23.7%)	20 (21.5%)	19 (20.4%)	22 (23.7%)	10 (10.8%)

Tables 4, 5 show the average scores of the participants for perceived levels of competence and perceived sexual health barriers. A simple additive method was used to calculate and convert the scores to a hundred-point scale. For perceived competence levels, the calculated scores were converted as follows: Poor or No competence at all (≤50), Moderate competence (51-75), and Good competence (>75). For perceived sexual health barriers, the calculated scores were converted as follows: Low or No barrier at all (≤50), Moderate barrier (51-75), and Extreme barrier (>75).

**Table 4:** Perceived competence and sexual health issues barrier scores domain.

Domains	N	Min	Max	Mean	SD
Perceived competence score	93	5.56	100.00	64.81	19.3
Sexual health issues barrier score	93	15.00	100.00	66.83	20.1

**Table 5:** Distribution of perceived competence levels and barriers in addressing sexual health issues (n = 93).

Domains		Count	%
Total		93	100.0
Perceived competence levels	Poor or No competence at all	23	24.7
	Moderate competence	40	43.0
	Good competence	30	32.3
Sexual health issues barrier levels	Low or No barrier at all	23	24.7
	Moderate barrier	39	41.9
	Extreme barrier	31	33.3

Nearly half of the participants (40, 43%) reported having moderate perceived competence levels, 30 (32.3%) had good perceived competence, and 23 (24.7%) had poor or no perceived competence concerning sexual health concerns. The mean score for perceived competence levels was 64.81, with a standard deviation (SD) of 19.3. Moreover, nearly half (39, 41.9%) noted moderate-level barriers, while 31 (33.3%) reported extreme barriers, and 23 (24.7%) reported low or no barrier regarding sexual health issues. The mean score for sexual health barriers was 66.83, with an SD of 20.1.

The correlation between perceived competence and sexual health issues barrier scores is presented in Table 6. This table reveals a significant negative correlation between perceived competence and sexual health issues barrier scores (r = -0.227).

**Table 6:** Correlation of perceived competence and sexual health issues barrier scores.

Correlations	Sexual health issues barrier score	
Perceived competence score	r	-0.227
	p-value	0.029
	N	93

Table 7 shows the correlation of perceived competence scores with the socio-demographics of the 93 participants. This table reveals a significant association between perceived competence scores and gender (p-value = 0.023) but no significant association with position (p-value = 0.924).

**Table 7:** Correlation of perceived competence scores with socio-demographics of 93 participants. GP: general practitioner. ^a^Significant using chi-square test at <0.05 level.

Demographics		Total	Perceived competence levels			p-value
			Poor or No competence at all	Moderate competence	Good competence	
Total		93	23 (24.7%)	40 (43.0%)	30 (32.3%)	-
Gender	Male	35	5 (14.3%)	13 (37.1%)	17 (48.6%)	0.023^a^
	Female	58	18 (31.0%)	27 (46.6%)	13 (22.4%)	
Position	Consultant	16	3 (18.8%)	8 (50.0%)	5 (31.3%)	0.924
	Board-certified/associate consultant	16	3 (18.8%)	8 (50.0%)	5 (31.3%)	
	GP	22	5 (22.7%)	10 (45.5%)	7 (31.8%)	
	Resident	39	12 (30.8%)	14 (35.9%)	13 (33.3%)	

Table 8 presents the correlation of sexual health issues barrier level scores with the socio-demographics of the 93 participants. This table reveals no significant association between sexual health issues barrier level scores, gender, and position (p-value = 0.472).

**Table 8:** Correlation of sexual health issues barrier level scores with socio-demographics of 93 participants. GP: general practitioner.

Demographics		Total	Sexual health issues barrier levels			p-value
			Low or No barrier at all	Moderate barrier	Extreme barrier	
Total		93	23 (24.7%)	39 (41.9%)	31 (33.3%)	-
Gender	Male	35	10 (28.6%)	16 (45.7%)	9 (25.7%)	0.472
	Female	58	13 (22.4%)	23 (39.7%)	22 (37.9%)	
Position	Consultant	16	2 (12.5%)	8 (50.0%)	6 (37.5%)	0.472
	Board-certified/associate consultant	16	6 (37.5%)	3 (18.8%)	7 (43.8%)	
	GP	22	6 (27.3%)	10 (45.5%)	6 (27.3%)	
	Resident	39	9 (23.1%)	18 (46.2%)	12 (30.8%)	

## Discussion

This cross-sectional study is a comprehensive analysis of the perceived competence and sexual health barriers among family medicine residents and primary care physicians of the National Guard in the Western Region of Saudi Arabia. Several main points from the results of the study are assessed below.

This study showed a general female predominance of 58 (62.4%) compared to 35 (37.6%) male participants, which is expected in this research area due to the higher number of female physicians compared to male physicians. Among these participants were 39 (41.9%) residents, 22 (23.7%) GPs, and both board-certified/associate consultants and consultants comprised 16 (17.2%).

In this study, scores for each level of perceived competence and sexual health barriers were assessed. Most participants revealed high levels of perceived competence; however, nearly a third of them (31, 33.3%) showed “quite poor” levels when treating male patients’ sexual problems compared to females, with only 12 (12.9%). In line with this study, El-Sakka [15] noted that despite the negative effects that sexual dysfunctions have on quality of life, males in the Middle East have limited access to treatment for these disorders, low treatment participation, and low treatment completion rates. The apparent discrepancy between accessible treatment and communication, as well as Middle Eastern cultural and gender conventions that could discourage treatment-seeking, are other profound issues. Furthermore, 46 (49.5%) answered "seldom" to "never" when exploring patient satisfaction with their sexual health. In agreement with this, El-Sakka [15] also noted that healthcare professionals, including GPs, frequently avoid discussing sexual concerns even when a problem may be suspected, despite the high frequency of sexual difficulties in the Middle East. Additionally, these practitioners frequently believe that they lack the expertise and abilities needed for these kinds of discussions.

Additionally, in this study, among the listed perceived sexual health barriers, gender differences were considered the most common barrier to addressing sexual health issues, with 44 (47.3%) reporting this. Personal discomfort was noted by 32 (34.4%), personal attitudes and beliefs by 31 (33.3%), and sexual health issues not being a priority in the appointment by 27 (29.0%). In line with this study, Alomair et al. [16] stated that some healthcare providers’ attitudes and conduct, such as rushed delivery and poor communication, act as barriers to Saudi women’s sexual and reproductive well-being. The researchers also stated that the barriers include a range of issues encountered in clinical interactions with patients, such as lack of availability of educational resources, lack of time and training, difficulties in maintaining privacy/confidentiality, and lack of counseling, which agrees with the study of Do et al. [17].

Moreover, the study found that nearly half of the participants, 40 (43%), answered that they had moderate perceived competence levels; 30 (32.3%) had good perceived competence, and 23 (24.7%) had poor or no perceived competence concerning sexual health concerns. The mean score for perceived competence levels was 64.81 with a standard deviation (SD) of 19.3. Additionally, 39 (41.9%) noted moderate-level barriers, while 31 (33.3%) answered extreme barriers, and 23 (24.7%) answered low or no barriers regarding sexual health issues. The mean score for sexual health barriers was 66.83 with an SD of 20.1. In line with this study, Memish et al. [18] also researched doctors in Saudi Arabia regarding their knowledge levels in treating HIV. The results showed poor knowledge levels, thus negatively affecting the development of any preventive intervention programs for HIV. This study also revealed a significant negative correlation between perceived competence and sexual health issues barrier scores (r = -0.227). This implies that high levels of barriers lead to low levels of competence, while high levels of perceived competence lead to low scores of sexual health barriers.

Additionally, our study revealed a significant association between perceived competence scores and gender (p-value = 0.023), which may be attributed to female predominance among participants. However, corresponding studies by Mandil et al. [19] and Subki et al. [20] stated that both genders (67% of men and 85% of women) reported that they would prefer a female doctor for obstetric and gynecologic care, contributing to higher confidence among female physician experience.

The limitations of our study included challenges in minimizing disruptions in the clinical setting, as the workflow was intense and the physicians were often busy, making it difficult to find suitable times for them to fill out the questionnaires. Additionally, some physicians were on leave, requiring us to track their schedules and arrange meetings with them to ensure their inclusion in the study. Traveling between multiple cities posed another challenge, as moving back and forth was time-consuming, limiting the time available for data collection at each site. Despite these obstacles, we managed to collect data effectively during working hours.

## Conclusions

This study highlights the moderate perceived competence and significant sexual health barriers faced by family medicine residents and primary care physicians in addressing sexual health issues in the Western Region of Saudi Arabia. Nearly half of the participants reported moderate competence levels, with gender differences, personal discomfort, personal attitudes and beliefs, and the lack of prioritization of sexual health issues during appointments emerging as significant barriers. These findings underscore the importance of prioritizing sexual health education, addressing systemic barriers, and improving healthcare providers' confidence and skills in managing sexual health concerns. By enhancing provider competence and reducing barriers, tailored training programs and supportive policies can improve the quality of sexual healthcare delivery. These results provide a foundation for future research and policymaking to address sexual health-related issues, particularly in culturally sensitive settings.
